# Childhood and adolescent schizophrenia and networking

**DOI:** 10.1192/j.eurpsy.2024.965

**Published:** 2024-08-27

**Authors:** S. Perez-Sanchez, I. Martin

**Affiliations:** ^1^Psychiatry, Los Arcos del Mar Menor Hospital; ^2^Psychiatry, Infante-Murcia Mental Health Center, Murcia, Spain

## Abstract

**Introduction:**

Reviewing the histories of patients with childhood and adolescent schizophrenia, on numerous occasions, traumatic factors with great emotional and social impact are observed, such as situations of mistreatment, abuse and emotional deprivation, where help is requested but resources are scarce and the approach is limited without work in network.

**Objectives:**

1. Analysis of the consultation pathways in adolescents with severe psychiatric and socio-familial symptoms and the time delay in optimal guidance. 2. Evaluate the services involved. 3. Evaluate the clinical control of treatment with aripiprazole.

**Methods:**

Retrospective observational analysis, 5 months, of prodromal symptoms of childhood psychosis and help-seeking pathways in a 13-year-old adolescent, as well as a description of the pharmacological approach and professional teams involved.

**Results:**

A 13-year-old adolescent who went to the emergency room with her mother for ingesting anti-cockroaches with self-inflicted intent. Reviewing his medical history, 4 serious autolytic gestures were detected in the last 2 months. Referred to Mental Health who did not attend.

On clinical examination, florid psychotic, with perplexity, self-reference, ideation of harm, language and behavioral disorganization. Auditory and kinesthetic hallucinations. A situation of neglect of the minor and abuse by the parents is detected; the risk had not been previously reported. Oral aripiprazole (up to 15 mg/day) was prescribed with good tolerance and progressive improvement in symptoms.

We worked jointly with Social Services, the Juvenile Prosecutor’s Office, Education and the hospital Psychiatry unit. In some of the resources the situation was already evident, the scope of their action was limited and delaying attention for months.

**Conclusions:**

Based on cases like this, we ask ourselves if psychiatrists are sensitized to the comorbidity of serious mental pathology and the existence of risk situations in minors and if they are oriented toward multiprofessional management. Networking provides us with an early diagnosis, improving the therapeutic approach.

**Disclosure of Interest:**

None Declared

